# Implementing ‘Closing the Gap’ policy through mainstream service provision: A South Australian case study

**DOI:** 10.1002/hpja.884

**Published:** 2024-06-02

**Authors:** Emma George, Matt Fisher, Tamara Mackean, Fran Baum

**Affiliations:** ^1^ School of Allied Health Science and Practice, University of Adelaide Adelaide South Australia Australia; ^2^ Stretton Health Equity, School of Social Sciences, University of Adelaide Adelaide South Australia Australia; ^3^ College of Medicine and Public Health, Flinders University Bedford Park South Australia Australia

**Keywords:** case study, health inequities, health services, leadership, policy

## Abstract

**Issue Addressed:**

The Australian government's ‘Closing the Gap’ (CTG) strategy has been implemented via multiple strategies. We examined CTG policy in early childhood within Southern Adelaide during the first decade of implementation (2008–2018) and critiqued the complexity and challenges of policy that is designed to promote health and well‐being of Aboriginal and Torres Strait Islander children but lacked Aboriginal control.

**Methods:**

A qualitative case study was conducted in Southern Adelaide, and we interviewed 16 policy actors from health and early childhood education sectors. Thematic analysis revealed key themes to show how policy had been implemented through mainstream structures.

**Results:**

The rapid roll out of the CTG strategy, the limitations of short‐term funding, cuts to Aboriginal health services, tokenistic consultation, and the mainstreaming of service provision were key features of policy implementation. The influence of Aboriginal leaders varied across implementation contexts. Participants advocated for services in health and education that are culturally safe to improve health of children, families, and communities.

**Conclusions:**

The implementation of the CTG strategy in Southern Adelaide was rushed, complex, and lacking Aboriginal control. This contributed to the marginalisation of Aboriginal leaders, and disengagement of families and communities. A more collaborative and Aboriginal led process for policy implementation is essential to reform policy implementation and address health inequity.

**So What?:**

Findings from this study suggest that policy has continued to be implemented I ways that reflect colonial power imbalances. Alternative processes that promote the recognition of Indigenous rights must be considered if we are to achieve the targets set within the CTG strategy.

## 1 BACKGROUND

A healthy start in life gives children the chance to thrive and to make a positive contribution to society.^1^ Hertzman and colleagues have shown that childhood experiences affect health in profound and long‐lasting ways.^2^, ^3^ The barriers and enablers of healthy child development are complex and interconnected. Colonisation has had far reaching destructive consequences for Indigenous people and communities globally.^4^ Policies resulting in the forced removal of children resulting in stolen generations were built upon assumptions of white superiority over Indigenous peoples,^5^ and lack of recognition of Indigenous rights.^6^, ^7^, ^8^ The damage done by Australian policies of segregation, marginalisation, and assimilation still impact on the lives of children today.^9^, ^10^ In 2023, the proportion of Aboriginal and Torres Strait Islander children aged 0–17 in out‐of‐home care was 43.7%, and increase of 3.7% since 2019.^11^ The trauma associated with the forced removal of children has significant impacts on social and emotional wellbeing and contributes to current inequities in health, education, employment, housing, and criminal justice.^5^ The ‘Footprints in Time’ longitudinal study on Australian Indigenous children^12^ reported Aboriginal and Torres Strait Islander children continue to experience, often as a result of disadvantage, much higher rates of major life events (e.g., death of a family member, financial stress, substance misuse and family separation) resulting in social and emotional difficulties, with research now showing detrimental long‐term effects on development.^13^ Policy reform and investment in early childhood is an important strategy for population health and health equity, and a range of other positive social outcomes.

The first decade of the Australian government's ‘Closing the Gap’ (CTG) strategy had two targets that prioritised early childhood^14^: to halve the gap in mortality rates for Indigenous children under five within a decade, and for 95% of all Indigenous 4‐year‐olds to be enrolled in early childhood education by 2025. These targets reflect the importance of both health and education. Both sectors are responsible for implementing early childhood policy and with the emergence of services such as Children and Family Centres under the CTG strategy, we note that policy has been implemented through intersectoral approaches. While the rate of child mortality dropped for Aboriginal and Torres Strait Islander children during this CTG strategy era, so too did the child mortality rate for non‐Indigenous children, and therefore, the gap widened. Child mortality for Aboriginal and Torres Strait Islander children was measured at 2 times the rate of non‐Indigenous children, 141 compared with 67 deaths per 100 000 for non‐Indigenous children.^14^ In addition, improvements in maternal health were not translated into improved health of children. Attendance in early childhood education for Indigenous children was high and recorded at 97.8%, but only 82% attendance was recorded for Indigenous students compared with 92% for non‐Indigenous students.

The research team's previous analysis of early childhood policies within the CTG strategy from 2008 to 2018 identified the inconsistent recognition of Indigenous rights in policy.^15^ During this time there were two clear iterations of policy which reflect changes in government from a Labour‐led era (Rudd‐Gillard‐Rudd 2007–2013), to the Coalition (Liberal and National) era (Abbott‐Turnbull‐Morrison 2013–2018). The policy analysis showed that Indigenous rights could be undermined or ignored when policy strategies prioritised mainstream service provision, promoted universal access to services, made assumptions reflecting colonial power structures, and focused on deficit narratives. Much of the CTG strategy in early childhood has been implemented through mainstream health and education services. Jongen, McCalman^16^ argued that both Aboriginal controlled and mainstream services have an important role in the provision of health services. The concept of ‘mainstream’ services in this context refers to government‐run services available to the general population as distinct from public or community‐controlled services run by and for Aboriginal people specifically. However, Freeman et al.^17^ found that Aboriginal people in their research were less likely to access mainstream early childhood health services due to fear of child removal and a lack of trust in the provider. Cooper^18^ argued that requiring Aboriginal and Torres Strait Islander people to access mainstream services when it is the only option available, subjects them ‘to increased levels of government control, surveillance and intervention in the name of addressing disadvantage and community dysfunction’ (p. 19).

The Close the Gap Campaign Steering Committee^19^ argued that while the CTG strategy has merit in attempting to address chronic disease, child and maternal health and other areas, there remains a lack of comprehensive action addressing the underlying causes of health inequity. This research aims to explore the way that the CTG strategy was implemented in early childhood through a mainstream model for service provision in the Southern Adelaide region during the first decade of the CTG strategy. A focus on policy implementation in a localised place can expose the impacts which government agencies' operational norms and practices have on Aboriginal communities.^20^ This research addresses the following research questions:

1. What was the experience of the ‘CTG’ strategy in early childhood in Shepparton Southern Adelaide (South Australia)?
2. To what extent are Indigenous rights prioritised and acted on within the ‘CTG’ strategy in early childhood?

### 1.1 Southern Adelaide case study

Southern Adelaide in South Australia was identified as an appropriate research case study during the planning of the Centre for Research Excellence on Social Determinants of Health Equity,^21^ knowing that the CTG strategy in early childhood has been implemented with different levels of community engagement, local leadership, and community control. The decision to focus on this case study was developed in consultation with Aboriginal and non‐Indigenous key stakeholders. The Southern Adelaide case study provided a platform to explore the complexity of local policy implementation through mainstream services.

Southern Adelaide is a metropolitan region spread over ~570 km^2^ with a growing population of 272 859 (across the Marion and Onkaparinga local government areas), where 1.7% identify as Aboriginal or Torres Strait Islander.^22^ The Kaurna people are acknowledged as traditional owners of the Adelaide plains, including Southern Adelaide. The Southern Adelaide Local Health Network provides health services across the region, including the Flinders Medical Centre, Repatriation General Hospital, Noarlunga Hospital, GP Plus Health Care Centres and Super Clinics, and sub‐acute and mental health services.^23^ Aboriginal‐specific health clinics operate at two sites in Southern Adelaide (Noarlunga and Clovelly Park) promoting health and well‐being through culturally appropriate services that are responsive to the needs of the community.^24^

The Taikurrendi Children and Family Centre (Taikurrendi) was newly established under the CTG strategy. The centre is embedded within the state education department. As a mainstream children's centre, it is open to all members of the community for preschool, occasional care, community groups, and additional parenting support and health services. Overall enrolment at the centre in the kindergarten program has increased from 41 children at the start of 2015 to 54 by the end of 2019,^25^ with attendance consistently over 85%. The number of Aboriginal children included in these statistics is not available.

The Christie Downs Kindergarten is located <2 km from Taikurrendi. Christie Downs Kindergarten is also part of the state education department, open to all families in the community, and has supported Aboriginal families for multiple generations. At the time of the study, they had similar enrolment numbers to Taikurrendi with 51 children enrolled at the end of 2018^26^ and while unpublished, there is consensus in the community that ~80% of the children at Christie Downs Kindergarten would identify as Aboriginal.

## 2 METHODS

This research adopted decolonising methodology.^27^, ^28^ The first author is a non‐Indigenous researcher and in an in‐depth reflection on her standpoint, they outlined the importance of decolonising transformation unlearning that challenged colonial ways of thinking and embraced opportunities for learning from Indigenous knowledge.^29^ This work was supported by a supervisory team that included an Aboriginal public health leader and researcher (third author), who challenged the first author to situate this research at the interface of knowledge.^30^ Local community engagement provided opportunities to build mutual respect and acts of reciprocity, consistent with principles of research at the interface of knowledge. Community engagement was appropriately tailored for the case study to reflect the Southern Adelaide context by connecting with local service provider networks and participating in community events. Data were gathered through in‐depth, semi‐structured interviews with policy actors. Participants were identified as leaders of health and education services in early childhood and through snowball sampling. An interview guide was developed with questions related to the work of the participant's organisation, partnerships, CTG targets and implementation, social determinants of Indigenous health, Indigenous rights, self‐determination, and Indigenous knowledge. Twenty‐three people in Southern Adelaide from health and education sectors were invited for interviews and 16 people agreed to participate, 9 of whom identified as Aboriginal or Torres Strait Islander. Interviews lasted between ~60 and 90 min, conducted at a convenient time and location convenient. This included a room within a public or office building, or a quiet café. Interviews were audio‐recorded and transcribed verbatim. Interview transcripts were coded using NVivo 11, where emergent, repeated patterns of meaning as well as differences were identified.^31^ Analysis was both inductive and deductive whereby themes emerged from the data, but also known ideas of social determinants of Indigenous health, self‐determination, and decolonisation were explored in depth.^32^

A workshop was facilitated in 2018 to present preliminary analysis of the data and seeks feedback and input from research participants. Five participants attended. This workshop enabled the research team to discuss interpretation of the findings and whether this was consistent with Indigenous ways of knowing. Discussion on data analysis focused on the following:

1. A complex and changing policy environment.
2. A regional approach—or ‘place‐based’ policy.
3. Aboriginal self‐determination in the region.
4. Social determinants of Indigenous health.
5. Child health and child rights.
6. Key messages to government.

A member of the research team took detailed notes at each workshop. Following the completion of the workshop, the team met to discuss any new insights and reflect on the contributions of all participants. Detailed minutes from these meetings informed the thematic analysis.

1. A final workshop was conducted in 2019 to discuss the overall results and seek feedback on opportunities for dissemination. Three participants attended. Rapport between participants (who were often known to each other) and trust extended to the research team enabled open, honest, and respectful communication. Discussion on the analysis to date focused on: Experiences of consultation
2. Understanding self‐determination
3. Impact of policy implementation on the workforce
4. Role of key stakeholders in policy implementation

Participants discussed each theme in detail and supported the findings of the research.

Informed consent was obtained from each participant at every stage of the project. The research has ethical approval from the Flinders University Social and Behavioural Research Ethics Committee (project number 6786), and the Aboriginal Health Research Ethics Committee (a sub‐committee of the Aboriginal Health Council of South Australia Inc (project number 04‐16‐697)). All information provided was treated in strict confidence, and every effort made to protect anonymity of participants throughout. Under recommendation from the Aboriginal Health Research Ethics Committee, in order to protect the anonymity of participants, participant connections to specific health and education sectors or organisations has been removed, and quotes in the results section are not linked to any identifying feature other than Aboriginality when necessary.

## 3 RESULTS

In this article, we present key themes conceptualised from the data.

### 3.1 The rapid introduction of the CTG strategy

The CTG strategy in Southern Adelaide was described by a participant as a wave of ‘unprecedented funding into Aboriginal health space’. Despite the initial optimism about new opportunities the CTG strategy offered, Aboriginal participants agreed with the sentiment of one participant who said, ‘it was flawed from the get‐go’, because of the sudden influx of funding and rushed implementation. One participant explained ‘with a rapid process you actually leave out key processes, key people, key structures. In order to get something out quickly, those things suffer’. Another non‐Indigenous participant expressed concern over the rapid roll out and said, ‘the money came in a bit like a freight train, and we had to, literally in a week's time, come up with a comprehensive state plan’. This was confirmed by another Aboriginal participant who explained the CTG strategy ‘just got chucked out there really quickly because you need to start spending it or you'll lose it’. On reflection, one participant suggested that ‘what really ended up happening was that people were putting in their pet projects as opposed to a strategic approach’ and that an opportunity had been missed to ‘take stock of what our data and our [evidence] is telling us about Aboriginal health’ which would have led to implementation in ‘areas of greatest need for the greatest impact’. Ultimately, all participants' comments indicated that the rapid roll out CTG policy in SA occurred without clear direction from the federal or state government.

### 3.2 The impact of short‐term funding

As a result of the rapid roll out, funding to organisations through CTG was short‐term which seemed to ‘really put Aboriginal led organisations on the back foot’. An Aboriginal participant explained ‘all we're doing now is going year to year and that's not commitment, that's just ad hoc funding and that stop‐start thing, does not work for our Aboriginal communities’. In addition, participants agreed that it was difficult to obtain long‐term support because ‘it's hard to make a case [for Aboriginal health] when outcomes are very long‐term, beyond everyone's contract, you know, beyond an electoral cycle’. Participants explained that the lack of long‐term support was not only seen in early childhood but across other sectors as well. In summary, the data showed that the imperative felt by State government actors, to roll out something quickly to take advantage of a sudden injection of Federal funding, prevented a more considered, community‐engaged process of policy planning to meet community needs.

### 3.3 Cuts to Aboriginal health services

At the same time as the rapid roll out of the CTG strategy, significant cuts to government Aboriginal health services were enforced. Within the state health department, the ‘Aboriginal health branch used to have 30 people in it; now it's got five’. Participants reflected on the cyclical nature of policy in Aboriginal health and one participant explained ‘they'll cut, then they'll set up an Aboriginal unit with resources because it's the flavour of the month… you go on and things start to roll, you'll get some consistency and then the next government will come in, and you'll roll back again’. They went on to say, ‘they'll cut, and it'll look completely differently to how it was – even if there's evidence to say things are working, still things get cut’. The significant cuts to Aboriginal health resulted in structural distance between local Aboriginal health services and health department leadership. One participant explained,

When I had a look at the restructured organisational chart it was [the Minister] at the top and Aboriginal health did not have a direct line of reporting anymore; they were about fifteenth on the ladder… second from bottom, and with no opportunity to speak… It was this convoluted and complex reporting process up to that level… the distance was significantly increased for Aboriginal people's voices to be heard.

All participants supported the ‘need for Aboriginal leadership throughout the policy process’. There was consistent promotion for ‘Aboriginal people writing, if not co‐designing policy… We can't be everywhere… but we can be concentrated on our Aboriginal business’. However, as one participant stated, it appeared that‐ there funding cuts were decided by ‘the executive [in the government Health Department who] have no experience working with Aboriginal people and they're not very up to date at all with Aboriginal health’.

Aboriginal participants expressed their frustration with the way that policy was implemented. As one participant argued:

We would have worked out a way to actually get collaborative support from key parts of the department to ensure that we had capacity to maintain [structures and services] … and then [the restructure] became terrible… I said I don't understand where you're coming from because what that shows, is you're actually ignorant… you don't do things for us without us… I never felt so marginalised… They saw us as being disgruntled, unhappy.

A non‐Indigenous participant also acknowledged the pain caused by significant cuts to Aboriginal health services. They explained ‘that's the heartache of it all… Once the funding ceased, numerous Aboriginal people lost their positions, programs no longer existed… Aboriginal people either became sicker, services closed or [programs] went to mainstream services which are culturally unsafe’. On the other side of the story, policy actors explained some of the cuts were a result of a lack of reporting where ‘on paper, [some programs] looked so bad that we could not possibly justify putting them forward’. This was ‘not necessarily because the people on the ground may have done a poor job, they just didn't give us ammunition to fight with’. They clarified that the lack of evidence to support programs was not a result of poor services or an inability to complete the work, rather the lack of reporting was due to excessing administrative demands putting excess stress on services. These reflections highlighted the ways that Aboriginal health workers made judgements about what was working that can be different to ways bureaucrats make judgements. Additionally, services may not have the resources to generate the data that bureaucrats supposedly need.

### 3.4 Perspectives on consultations related to early childhood services

Participants expressed frustration with the process of how consultation occurred around CTG funding for Aboriginal early childhood services in Southern Adelaide, as well as the underlying assumptions or intentions. Experiences of consultation were described as ‘tokenistic’. Two participants referred to consultation as a ‘tick the box’ activity of policy implementation. A non‐Indigenous participant said, ‘for me it was a tokenistic approach, getting an Aboriginal person to sit at the table, “yep, we've ticked that box” and the biggest anxiety for me was the lack of Aboriginal consultation in that process, the lack of community people that were brought to the table to truly say “this is what we need for the south”’.

Participants explained the tokenistic and frustrating experiences of consultation led to community conflict, particularly about the process of establishing Taikurrendi and the lack of resources made available to Christie Downs Kindergarten. On one hand, some participants felt that they knew Taikurrendi was always going to be embedded with a government department and therefore located at the site of the local primary school. This was described as a ‘non‐negotiable’. Other participants felt this was never clearly outlined and therefore they felt that Taikurrendi never really belonged to the community in the way that Christie Downs Kindergarten had belonged to the community for generations. One participant explained that the community lack of awareness of the ‘non‐negotiables’ meant that it was impossible for community to feel part of the process and have ownership. As a result, ‘people are holding on to those expectations’ and then community feel let down by workers who they trusted. One participant suggested that

there was never, ever a thought of [Taikurrendi] being an Aboriginal kindy even though it was [established] with Aboriginal money… The spirit of the agreement was about health and education coming together to have the best outcomes for Aboriginal kids, [but] it's a state kindy’. Another participant explained ‘it's more mainstream that it is Aboriginal’. A third participant agreed with this sentiment and explained ‘I think the initial concept of Taikurrendi [as an Aboriginal children's centre] was pretty special and we missed that opportunity.

The other side of the story is that the ‘non‐negotiables [for establishing Taikurrendi] were always there’, and the benefit of situating the centre within a government department is a financially sustainable children's centre with an Aboriginal focus. This sustainability is a very different experience to the cuts seen in the Aboriginal health sector. One non‐Indigenous government participant argued that consultation on Taikurrendi was

not just a bit of hit and miss, it was really well thought out, authentic, progressive, a slow walk to what we thought would be best for community… in terms of maximising outcomes for all our kids but particularly our Aboriginal and Torres Strait Islander kids at the end of the day.

They claimed that community assumptions that Taikurrendi would be just for Aboriginal and Torres Strait Islander children and community was a ‘misconception’, which in hindsight, meant that from the outset, consultation was always going to appear tokenistic. These assumptions, misconceptions, unmet expectations, and frustration led to some Aboriginal families avoiding the centre, whereas others took ‘a couple of years’ to begin to feel comfortable visiting the site.

Our results are suggestive of inadequate consultation processes that contributed to conflict within the community, adding to a psychological toll of feeling unheard during policy implementation.

### 3.5 Implications of a mainstream model

Participants expressed disappointment with services being embedded within the mainstream. One participant explained ‘you see things get cut and the next thing you know, they're given to a mainstream service with no Aboriginal faces in there, no Aboriginal knowledge in there… You can't do that without Aboriginal staff, Aboriginal leadership, Aboriginal advice… it'll just go backwards’. One participant explained that the local health clinic ‘just doesn't feel like it's an Aboriginal health clinic, it just feels like a mainstream health service – my experience going in and out is a bit like it could be any other service’. Participants identified limitations of mainstream service provision because ‘for Aboriginal people, wherever they go, they don't feel like it is for them’. One participant explained ‘the push to try and get people out into the mainstream is all well and good if that worked for everybody… [but] one size doesn't fit all’. One participant compared mainstream service provision to cultural blindness, and it ignores ‘a responsibility to fix the system that broke people’.

Aboriginal participants linked the mainstreaming of services to the lack of progress in closing the gap in health. One participant argued ‘I can't see any gaps being closed, I really can't… because when you look at it from how we are, an Aboriginal [targeted] service in the mainstream, there are policies and procedures that restrict us’. Another participant explained that they were directed to implement policy in specific ways according to national or state priorities and were unable to respond to local needs. They stated ‘what would work for Southern Adelaide, what would work for Northern Adelaide, it's different and the needs are different, and the communities are different… We threw all this money at [the gap] and it hasn't gone away’.

### 3.6 Experiences of racism in policy implementation

Both Aboriginal and non‐Indigenous participants highlighted that ‘mainstream services are culturally unsafe and [community] don't feel as if they are being heard or understood. They are confronted with racism and discrimination every day. It's just so much part of their lives that it makes them sick’. An Aboriginal participant argued that racism ‘is felt every day by Aboriginal people across the country. The judgement, the racism, the stereotypes’. The outcome of ‘cultural incompetency’ within the heath system, is that Aboriginal people ‘just don't go’. This was described as a barrier for community to access and receive health services.

In addition, participants described a secondary impact of racism on workers who implement CTG policies. An Aboriginal participant felt discouraged and overwhelmed by the ‘countless times’ clients were faced with unsafe practices and they explained with tears in their eyes, ‘there's nothing we can do about it except encourage the client to make a complaint, fill out of form, which they don't do, and they never do’. In response to these experiences of racism, Aboriginal participants reported that they had approached non‐Aboriginal health sector leadership to try and explain ‘when I see things, you don't see them the same as me’ and then they encouraged leaders to

actually be really mindful about unconscious bias in policy development and decision making… you have a responsibility that when you're writing policy and implementing policy, it actually needs to benefit the people that least look like you

Participants shared experiences of racism and a lack of cultural safety that led to fear of child removal, fear of assimilation and fear of the health system. One participant explained, ‘most Aboriginal people are scared to go to the doctor’. The ‘fear of assimilation’ was embedded within comments on the pressure to conform to a mainstream model of policy implementation and practice. ‘I worry about assimilation. I think we can work together in this community, in this country, but if you take their right to be Aboriginal away from them, then what have they got left?’ Participants reflected that the fear of assimilation weighs heavily on the Aboriginal community who are fighting for their cultural identity, as an essential part of their ‘being, belonging and becoming’. The fear of accessing health services and assimilation was described as a factor influencing engagement in a range of services, including early childhood education, ‘services that really provide critical support at a time when families are in the most vulnerable situations’.

One participant identified a way to begin to combat racism involved the ‘visibility’ of Aboriginal culture as an essential feature in health and education services. They maintained that when Aboriginal staff and culture are present, community are more likely to engage in services. For example, ‘the fact that when you walk through the gates you start being presented with Aboriginal design you start seeing an Aboriginal presence in a place. You go “this is me”’.

### 3.7 Impact of CTG policy implementation on the workforce

Insecure employment was highlighted as a major issue for the CTG workforce. At the time of an interview, one Aboriginal participant had only 17 days left on their contract without knowing if their program was going to be refunded. They described the uncertainty as a considerable source of stress and that it results in losing senior staff to more secure employment. They explained ‘we lose a lot of passion and drive that these people carry. They come into this job with the intention, the motivation, the initiative, the passion to want to make this change, but are constrained’. Another Aboriginal participant added that recruiting Aboriginal staff to grant funded positions was challenging and that the insecurity ‘is actually detrimental to Aboriginal health… it's just an absolute nightmare for us so sustainable funding is a big thing here’.

Aboriginal participants who have stayed involved in policy implementation, reflected on their motivation to be there for ‘our mob, [they] need us’. This sense of responsibility and purpose was reflected in comments such as

they need us to help them through navigating services, need us to carry them emotionally. We are the ones that are standing there saying ‘you have options when [the department] are coming to take your children, do you know you could ask these questions? When police stop you, do you know you can ask these questions?

The role adopted by this participant was described as ‘like being a stick in the mud’. The image they portrayed was that they provided stability in both a fluid workforce and a community in which power imbalances created vulnerabilities.

Importantly, Aboriginal staff were described as crucial to successful CTG policy implementation. There was consensus among all participants that ‘Aboriginal people prefer Aboriginal workers’. One participant explained that ‘employing Aboriginal staff members is part of building community capacity and it incorporates culture into the mainstream society. Transmission of cultural knowledge, languages, and cultural experience through activities and excursions; this is what happens when we employ Aboriginal people’. Aboriginal staff who implement policy in community are seen as ‘positive role models’ and they play a vital role in the delivery of culturally safe services. One participant explained ‘our staff are part of the community, so they know the [children and families] coming in. They know that they're going to be welcomed when they come in, that's so important. To make a service accessible is just so, so important’. These participants working among community reflected on the importance of relationships in ensuring services remain welcome and safe. Aboriginal staff ‘know what's going on… whether there's been a funeral two days ago, or whether someone is dying in hospital… Community dynamics really come into play with how you come across and pitch something… having a fun day when you've just lost a significant elder the day before – no one's going to come, plus you might actually damage your relationship with community because it's disrespectful’.

### 3.8 Understanding Aboriginal childhood

Participants shared a vision for an Aboriginal childhood. They advocated for ‘our children to be proud of who they are’, ‘to be connected to family, connected to culture, connected to identity’, and not to miss out on anything’. Indigenous language and understanding cultural practice were seen to be a critical part of cultural identity but ‘it was taken away from us to assimilate… That's not a good enough reason to strip somebody of their identity’. This participant went on to provide an insight into cultural practice and connection to country. They said ‘In Aboriginal culture, when Aboriginal people go hunting, they would sing to the bush because that's their connection to land. That's a connection to all that around them are living. Children need to feel that connection and our ancestors need to hear them, so they need that’. This reflects Indigenous knowledge and reveals a way of understanding of life and health that participants described as inconsistent with mainstream models of health and education.

Another example of understanding Aboriginal childhood was seen in the descriptions of raising small children. An Aboriginal participant explained their experience was that ‘Aboriginal babies are more likely to get held upright, so their necks get stronger earlier… they might be able to be settled by more different people, and they might be passed around more, but it's safe’. They went on to share that ‘you don't make kids cry so putting a kid in the bedroom and shutting the door and leaving them crying, I would get flogged for that. My mother would come in or my family would come in and go ‘what the hell are you doing?’ and pick baby up’. Participants expressed their frustration with the way they see Aboriginal families being judged on their parenting by expectations and norms that are more aligned to a western knowledge of raising children. One participant explained that government workers can come in an Aboriginal family home to assess living conditions and child safety. They ‘look around the house, have they got this, have they got that, have they got a baby's room set up and are they ready? It's all on a white lens’. They explained this is not just a result of individual bias, but it reflects a system of ‘education, experience, what they've been told to look for’ that does not understand Aboriginal ways of knowing, being, and doing. They shared, ‘in the old days on welfare you walked into a house, if there's no food in the cupboards the kids get taken away. Yeah, but it's an off‐pay week we go to Aunty's to eat, and then on our pay week, she'll come to us, and her cupboards will be empty’. A non‐Indigenous participant observed that Aboriginal families ‘just shut their mouth until you get the hell out of that house and off you go’. Another non‐Indigenous participant reflected on the different ways of raising children, and that this is not well understood by mainstream services even though ‘Aboriginal culture has survived 60 000 plus years, to do that you have to have got raising children right, so we have so much to learn [from Aboriginal culture and knowledge] really’.

Participants also identified that one of the biggest barriers to supporting Indigenous rights and understanding Aboriginal childhood is the removal of children from Aboriginal families. One participant explained ‘when you look at what actually transpires in statistics, there are incredible rates that Aboriginal children are being taken away from their families, so we don't do enough around prevention, early intervention in supporting vulnerable families, particularly Aboriginal families’. This was recognised by participants as a gap within the CTG strategy because they were no targets specifically measuring the numbers and experiences of Aboriginal children who are removed from their families, or investment in prevention of child removal through CTG policy implementation.

### 3.9 Understanding power in policy implementation

Participants reflected on the position of Aboriginal people as ‘other’ within policy and implementation processes. One participant acknowledged that ‘for too long we've been on the periphery of society’. Another participant argued ‘we're always bastardising every Aboriginal experience to be a negative experience, you know, always a deficit model approach to things’. One participant argued ‘the white way of doing things is the dominant way of doing things… especially in government’. Participants described power imbalances as a barrier in policy implementation that is difficult to overcome because ‘you're born into that kind of thing and unless you are doing some really deep, critical engagement to decolonise’ the barrier to equitable policy implementation remains. One participant stated that ‘I think people in Australia are happy with the status quo, they like Aboriginal people to be in a certain place in a certain way and have a certain level of control… I think people do like that, and they're comfortable with that’.

Participants also discussed the government's position of power over Aboriginal people as open to challenge. They advocated for a shift in power to occur so that there is ‘unlearning about how to write policy’. One participant argued that political positions as well as ‘reasonings of wanting to help’ influence policy and implementation. Without reflection or unlearning, policy actors ‘come in and out, if you're only in it for your own selfish reasons, then you generally do more harm than good’. Aboriginal participants recognised that they did not want to rely on the ‘goodwill’ of non‐Indigenous leaders in policy anymore. Participants explained that the goodwill or good intentions of non‐Indigenous leaders in health and education can be eroded because too often, Aboriginal health ‘is [put in] the too hard basket, and so they shy away from it because they don't have any knowledge… Like I said, you can't expect people to make good decisions and do the right thing if they don't have the knowledge and the tools to be able to do it’.

## 4 DISCUSSION

This research highlights the multiple ways in which the perceived imperatives of government agencies and actors played out in policy implementation with different levels of influence from Aboriginal leadership and engagement. To answer the research questions, we discuss the experience of policy implementation in Southern Adelaide, and the inconsistent ways that Indigenous rights are prioritised and acted on, depending on the influence of policy actors.

Since colonisation, Indigenous ways of knowing, being, and doing, have been considered illegitimate by non‐Indigenous power holders and Indigenous knowledge is therefore dismissed by mainstream discourses as being inferior.^33^, ^34^ As a result, policy is written is ways that privilege the dominant majority culture in a way that is unquestioned,^35^ maintaining the status quo of colonising dynamics within policy structures. The prioritisation of mainstream services for policy implementation missed the opportunity to prioritise Aboriginal understanding of health and self‐determination and addresses power imbalances.

Power is often analysed through terms of repression or liberation however this fails to embrace greater complexities of power. Australia has been built on a patriarchal white sovereignty even though Aboriginal sovereignty has never been ceded.^36^ A dominant expression of power has ‘allowed white colonists to treat Indigenous people as sub‐human, enabling them to appropriate Indigenous lands in the name of patriarchal white sovereignty’.^36^ Historical records of Indigenous sovereignty claims have detailed the way that racism is embedded into historical, political, and legal issues of sovereignty.^8^, ^37^, ^38^, ^39^ Aboriginal and Torres Strait Islander people have been denied the rights and protection generally afforded to Indigenous peoples in other parts of the world.^40^

In 2007, the United Nations' ‘Declaration on the Rights of Indigenous Peoples’ recognised dignity, wellbeing, and rights of the world's Indigenous people, in addition to basic human rights. Controversially, Australia, New Zealand, Canada, and the United States of America all initially voted against the adoption of the Declaration in 2007.^41^ Their opposition was focused on the term ‘self‐determination’ and the Australian Liberal Prime Minister at the time, John Howard, preferred a focus on ‘mainstream Australia’.^42^ Moreton‐Robinson^43^ argued that the Howard government moved away from Indigenous rights by focusing on ‘practical reconciliation’ and mutual obligation contracts which monitored and disciplined Indigenous subjects (p. 67). The policies and approaches of the Howard government years have been criticised as a return to principles of control and assimilation.^44^ The Australian Human Rights Commission^45^ reported it was not until 2009 that a Labour government under Kevin Rudd pledged its support to the Declaration. McLoughlin^44^ argued that government policies implemented since the Howard era are yet to deliver promises of self‐determination for Aboriginal people. Existing policies on Indigenous rights appear to be incompatible with the Declaration at beast, or a violation of rights at worst.^46^ Lavoie and Dwyer^47^ highlighted that without recognition of Indigenous sovereignty, self‐determination is not a priority for the Australian government, and it may require constitutional reform to fully address issues of governance and health equity. Treaties between Indigenous Peoples and settler states are a key factor that facilitates or obstructs the exercise of Indigenous sovereignty and Australia is yet to establish a treaty between Aboriginal and Torres Strait Islander people and the Commonwealth Government.^48^ The absence of a treaty means that Indigenous rights are unprotected in policy.^49^ The inconsistent recognition of Indigenous rights in policy^15^ means that policy can be implemented in flexible ways, through both mainstream and Aboriginal controlled sectors, leaving policy actors responsible for influencing implementation processes, and vulnerable to the pressures bearing down on them through power imbalances.

The flaws of CTG policy implementation were addressed to some extent by the influence of policy actors in the education sector who held steadfast to the need to promote the health and education of Aboriginal children. For example, programs facilitated through Taikurrendi targeted Aboriginal children and families, and the work of Christie Downs Kindergarten has continued despite not accessing the same CTG funding for their programs. The discretion demonstrated by Taikurrendi staff to build a centre with a focus on Aboriginal children, while located within the mainstream education department served as a way of implementing policy at a community level, where citizen interests are represented to some extent. However, according to our analysis, because Taikurrendi was not established as a community‐controlled service as was initially expected by some of the local community, the provision of culturally safe services is reliant on the influence of centre leaders, rather than more fully through a community‐controlled, self‐determining model.

The Southern Adelaide early childhood services for Aboriginal children are locked in to the mainstream and yet both Taikurrendi and Christie Downs Kindergarten have continued to prioritise education as an essential way for children to develop a strong sense of identity, connection to country and kinship,^50^ essential for the social and emotional well‐being of Aboriginal children.^51^ This highlights the significance of the influence of leaders in early childhood services at Taikurrendi and Christie Downs Kindergarten, who are committed and positioned to contribute to the cultural health and well‐being of Aboriginal children despite the many barriers they face in a mainstream model of service provision.

In contrast, policy actors in the health sector were unable to influence the way that policy was implemented to the same extent. It is therefore not surprising that participants described the lack of cultural safety in mainstream health services and experiences of racism. Paradies^52^ said racism permeates the very fabric of contemporary Australian society. Macedo et al.^50^ identified that racism towards Aboriginal people begins in childhood and leads to anxiety, depression, aggression, social and emotional difficulties, lower levels of self‐esteem, and other physical, social, and emotional problems. We argue that systemic racism is prevalent in this case study which highlights the drawbacks of mainstreaming, marginalisation of Aboriginal leaders, and tokenistic consultation in policy implementation.

One final point for discussion is the need to reframe the deficit narrative surrounding Aboriginal children and families. Bond and Singh^53^ argued that the CTG strategy is based on a form of bias, by being focused ‘disproportionately on the behaviour of individuals, suggesting that health inequalities are a product of Indigenous lack, morally and intellectually, rather than socially determined’ (p. 198). Across health and education, dominant early childhood development discourses are generalised, discounting socio‐cultural and historical contexts.^54^, ^55^ The invisible norms by which Aboriginal and Torres Strait Islander people and the CTG targets are measured to uphold a principle of ‘normalisation’,^56^ and Aboriginal people are continually represented as the problem.^57^ This deficit framing has been strongly criticised in the literature.^53^, ^58^, ^59^ Analysis of the deficit discourse by the Lowitja Institute highlighted that a reductionist deficit narrative distracts from the underlying reasons that lead to health inequities.^60^ An alternative approach to policy would align with decolonisation and community control. Fredericks^61^ argued that decolonising policy requires Aboriginal and Torres Strait Islander people to have control and participate in decision‐making, administrative process, and service delivery. This would shift the narrative from Aboriginal people being seen treated as objects of policy or problems to be solved, to stakeholders in policy with better control over health outcomes.

In the years since that first decade of the CTG strategy, the policy underwent a ‘refresh’ and 19 new targets have been set.^62^ Targets that specifically relate to early childhood include:

- Target #2. Children are born healthy and strong: By 2031, increase the proportion of Aboriginal and Torres Strait Islander babies with a healthy birthweight to 91%.
- Target #3. Children are engaged in high quality, culturally appropriate early childhood education in their early years: By 2025 increase the proportion of Aboriginal and Torres Strait Islander children enrolled in Year Before Fulltime Schooling (YBFS) early childhood education to 95%.
- Target #4. Children thrive in their early years: By 2031, increase the proportion of Aboriginal and Torres Strait Islander children assessed as developmentally on track in all five domains of the Australian Early Development (AECD) to 55%.
- Target #12. Children are not overrepresented in the child protection system: By 2031, reduce the rate of over‐representation of Aboriginal and Torres Strait Islander children in out‐of‐home care by 45%.

The policy rhetoric reflects a commitment partnership with people and communities and learning from past mistakes to avoid ongoing cycles of disadvantage and inequity. Annual reporting requires government agencies collaborate with Aboriginal and Torres Strait Islander peak partners to monitor progress and plan for future action. While the narrative has moved away from a deficit lens, we are not on track to achieving the new targets. The most recent government report^62^ reveals that for Target #2, there has been some improvement but we are not on track. For Target # 3, there has been good improvement and reports suggest we are on track to meet this target. For Target #4, the situation is worsening, and we are not on track. For Target #12, again the situation is worsening, and we are not on track.

The government report that significant investment has been made into strengthening engagement with the Secretariat of National Aboriginal and Islander Child Care (SNAICC) through the Early Childhood Care and Development Policy Partnership.^63^ This partnership supports shared decision making on early childhood education and care, maternal and child health, and child protection and families. One key initiative is the Early Years Support Program that is founded on self‐determination and community control. This is an important shift from past policies that prioritise mainstream services. In 2024, it is reported that program supports 42 Aboriginal Community Controlled Organisations, but none of them are located in South Australia.

To address the increasing representation of Aboriginal and Torres Strait Islander children in out‐of‐home care, several commitments have been made to end violence and abuse against women and children. Until evaluations of these types of programs are conducted, it is too early to determine whether they will meet objectives. There remains a risk, that new programs will repeat, the mistakes of the past and fail to address deep seated wounds stemming from racism, ongoing colonisation, and power imbalances.^57^ Participants in this research who were all working in mainstream services agreed that superficial solutions cannot address the underlying causes of health inequity in Australia.

We argue that in order to meet all the new targets within the CTG strategy, policy must reflect, recognise, and act on Indigenous rights, including self‐determination. The Coalition of Peaks^64^ argued that mainstream service providers must be held accountable and do much more to improve the health of Aboriginal and Torres Strait Islander people and communities. This includes tackling systemic racism, promoting cultural safety and transferring power and resources to communities, and Aboriginal controlled organisations. Federal, state, and local governments must trust Aboriginal leaders who know what they are doing, know their communities, and work in culturally safe ways. Maternal and child health, education, family services, and child and youth services (among others) are sectors identified by the Coalition of Peaks^64^ that require strengthening through development of community‐controlled services, and we advocate that new programs need to be rolled out nationally in every state. Our research showed that stable national, state, and local partnerships with these services should inform policy development and implementation. The findings from this research support funding being targeted and long‐term for the implementation of the CTG strategy to promote health and education outcomes for Aboriginal children, families, and communities. The insecure, short‐term, prescriptive, and fragmented funding that have been prominent features of the CTG strategy in the past must be discontinued. Long‐term funding for community‐controlled services and other services at the local level in health and education is essential to ensure that policy is implemented in ways that are anti‐racist, respond to local needs and improve health of children, families, and communities.

### 4.1 Limitations

Not every policy actor within early childhood responded to the invitation or participated in the study. However, living in the Southern Adelaide region allowed researchers to participate regularly with the community and develop a deep understanding of how policy was implemented.

## 5 CONCLUSION

This Southern Adelaide case study revealed the complexity and challenges of implementing the CTG strategy in early childhood through a mainstream model. Policy actors in health and education advocated for services led by Aboriginal people with an Aboriginal focus which are culturally safe and much needed to improve the health of children, families, and communities. However, the rapid roll out of the CTG early childhood strategy, the short‐term funding models, cuts to Aboriginal health services, flawed processes of consultation, and the issues associated with a mainstream model of service provision reveal the need for reform in policy implementation structures and processes. The diverse nature of Aboriginal and Torres Strait Islander peoples across Australia means that diverse actions are essential in policy implementation but there should be a way for the innovative responses to policy to go back up the chain to the policy makers. There will continue to be a gap in policy and inconsistent action for Indigenous rights without recognition of Indigenous sovereignty.

## FUNDING INFORMATION

This work was supported by the NHMRC Centre of Research Excellence in the Social Determinants of Health Equity: policy research on the social determinants of health equity (APP1078046). The Author received a PhD scholarship funded by the NHMRC which was topped up by Flinders University.

## CONFLICT OF INTEREST STATEMENT

Prof Fran Baum is an Editorial Board member of HPJA and a co‐author of this article. To minimise bias, they were excluded from all editorial decision‐making related to the acceptance of this article for publication.

## ETHICS STATEMENT

Ethics approval for this case study research was granted by Flinders University Social and Behavioural Research Ethics Committee: project number 6786 and the Aboriginal Health Research Ethics Committee (a sub‐committee of the Aboriginal Health Council of South Australia Inc: project number 04‐16‐697).

## Data Availability

Research data are not shared.
